# Supplementary material for: Follow-up lung ultrasound to monitor lung failure in COVID-19 ICU patients
Source: PLoS One. 2022 Jul 14;17(7):e0271411. doi: 10.1371/journal.pone.0271411 (PMC9282475; doi:10.1371/journal.pone.0271411)
Supplement: S1 Table — (PDF) [file pone.0271411.s001.pdf]

S 1 Table: Respiration and Ventilation in correlation with LUS

LUS /parameters of ventilation, day of mechanical ventilation	Pearson	p-value
**LUS (points)/p_aO2(mmHg), d1**	-0.327	0.147
**LUS (points)/p_aO2(mmHg), d7**	0.022	0.933
**LUS (points)/p_aO2(mmHg), d15**	-0.247	0.439
**LUS (points)/p_aCO2(mmHg), d1**	-0.016	0.945
**LUS (points)/p_aCO2(mmHg), d7**	0.364	0.150
**LUS (points)/p_aCO2(mmHg), d15**	0.834	**0.001**
**LUS (points)/FiO2, d1**	-0.117	0.624
**LUS (points)/FiO2, d7**	0.591	**0.033**
**LUS (points)/FiO2, d15**	0.827	**0.002**
**LUS (points)/p/F-ratio (mmHg), d1**	-0.234	0.322
**LUS (points)/p/F-ratio (mmHg), d7**	-0.723	**0.005**
**LUS (points)/p/F-ratio (mmHg), d15**	-0.861	**0.001**
**LUS (points)/PEEP (mbar), d1**	0.283	0.214
**LUS (points)/PEEP (mbar), d7**	0.495	**0.043**
**LUS (points)/PEEP (mbar), d15**	0.597	0.090
**LUS (points)/P_{plat}(mbar), d1**	0.376	0.093
**LUS (points)/P_{plat}(mbar), d7**	0.617	**0.008**
**LUS (points)/P_{plat}(mbar), d15**	0.625	0.072
**LUS (points)/ Vt (mL), d1**	-0.407	0.067
**LUS/ Vt (mL), d7**	-0.426	0.088

**LUS/ Vt (mL), d15**	0.493	0.177
**LUS/Compliance, d1**	-0.374	0.095
**LUS/Compliance, d7**	-0.572	**0.016**
**LUS/Compliance, d15**	-0.028	0.942
