# Supplementary material for: Follow-up lung ultrasound to monitor lung failure in COVID-19 ICU patients
Source: PLoS One. 2022 Jul 14;17(7):e0271411. doi: 10.1371/journal.pone.0271411 (PMC9282475; doi:10.1371/journal.pone.0271411)
Supplement: Attachment — Submitted filename: Response to Reviewers.rtf [file pone.0271411.s003.rtf]

Dear Editor, Dear Editorial Board Members, Dear Reviewers, 
Thank you for handling and reviewing our manuscript. We found the reviewers´ comments very helpful and adapted the manuscript accordingly. Please find a point-by-point response below.
Best regards, 
Michaela Barnikel and Stephanie-Susanne Stecher (on behalf of all co-authors)

Reviewer 1
Overall the study is clear but the hypothesis of the study is not new and the design of study is poor. The authors perform follow-up Lung ultrasound in patients on ICU to explore LU as a tool for monitoring lung failure caused by SARS-CoV2. Furthermore, they evaluate the difference in LUS over the course between invasive and non-invasive ventilated patients. In my opinion the study did not add relevant information to the current knowledge in this specific field of research and it could be limited interest by the readers. Overall confirmed results previously published by other group of research. The authors should clarify the novelty of the study.
Response: Thank you for this remark. We agree that numerous studies deal with lung ultrasound, especially during the pandemic. However, while performing our survey in 2020, lung ultrasound was just established in COVID-19 ICU patients. And as far as we know there are still only a few papers dealing with follow-up lung ultrasound in mechanical ventilated COVID-19 patients. Most of them handle lung ultrasound as an initial assessment of lung failure but not in follow-up. And so, we feel confident that our results are of interest to the readers of the PLOS ONE Journal. Exemplary, we listed some of the topical papers:  
Vetrugno L, Bove T, Orso D, Barbariol F, Bassi F, Boero E, et al. Our Italian experience using lung ultrasound for identification, grading and serial follow-up of severity of lung involvement for management of patients with COVID-19. Echocardiography. 2020;37(4):625-7.

Hoffmann T, Bulla P, Jodicke L, Klein C, Bott SM, Keller R, et al. Can follow up lung ultrasound in Coronavirus Disease-19 patients indicate clinical outcome? PLoS One. 2021;16(8):e0256359.


Methodologically; please clarify who performed the analysis of US and perform analysis in terms of inter and intra variability. 
Response: We have now clarified in our manuscript that only one senior physician with expertise in lung ultrasound supervised the performance of lung ultrasound and scored the images. A randomly selected number of ultrasound examinations were blinded and examined by another senior physician to obtain reliable results. We have now added a Bland Altman analysis for performing analysis in terms of inter variability. 

Furthermore should be clarified how do you set the mechanical ventilation in particular regarding the PEEP selection. 
Response: We thank the reviewer for broaching this issue. In our study cohort, best-PEEP selection was handled by performing PEEP trials by arterial blood gas analysis at different PEEP levels. We did not define best-PEEP by evaluating lung ultrasound when conducting this study. 

How do you manage the LU evaluation in case of pneumothorax during length of stay.
Response: Thank you for this mindful remark. While performing lung ultrasound, sonographic signs of pneumothorax, e.g., lack of lung sliding, lack of B lines, and barcode sign, were considered throughout. X-ray of the thorax was amended in case of suspected pneumothorax by sonography. However, there was no case of pneumothorax. We have now added this information to our manuscript.


Reviewer 2
The problem described in the paper is actual. The diagnostic tools for monitoring the patients with  coronavirus disease are in demand. The point-of-care lung ultrasound is promising because it is radiation free, comparatively available. Authors provided an adapted protocol for severe patients due to their limited positioning options. The research shown that the lang ultrasound can be a useful tool to monitor lung failure in COVID-19 ICU patients. The dynamics of lung ultrasound score is correlated with progression and regression of respiratory failure. The manuscript is structured, written in clear English. Raw data is not provided in manuscript and supporting materials, only mean values and standard deviations are given. Statistically computations have been performed appropriately.
Response: We thank the reviewer for dealing with our manuscript. We have now provided raw data in an excel-sheet. Please take heed of the attachment file.
