# Supplementary material for: Follow-up lung ultrasound to monitor lung failure in COVID-19 ICU patients
Source: PLoS One. 2022 Jul 14;17(7):e0271411. doi: 10.1371/journal.pone.0271411 (PMC9282475; doi:10.1371/journal.pone.0271411)
Supplement: Attachment — Submitted filename: Response to Reviewers .rtf [file pone.0271411.s004.rtf]

Dear Editor, Dear Editorial Board Members, Dear Reviewers, 
Thank you for handling and reviewing our manuscript again. We found the reviewers´ comments very helpful and adapted the manuscript accordingly. Please find a point-by-point response below.
Best regards, 
Michaela Barnikel and Stephanie-Susanne Stecher (on behalf of all co-authors)

Reviewer 1
The authors satisfied partially my previous comments and in my opinion the novelty of the study remain poor. However, I appreciate the efforts of the authors in orders to improve the quality of the paper. The authors reported that the novelty of the study regarding the follow-up lung ultrasound in mechanical ventilated COVID-19 patients. In my opinion, this is not clearly express in the text and I suggest to avoid confusion about the terms follow-up and the tool useful “to assess the progression or regression of respiratory failure.” Furthermore, the second aim is related to the first outcome. I strongly recommend to revise carefully it in according to the design of the study. In other words, I think that the study would like to evaluate the progression of the severity of disease and consequently assess if the LUS score could be useful to help in the decision of intubate or not intubate. This is the major point that should be addressed by the authors.
Response: We thank the reviewer for revealing this remarkable issue. We have now adapted our manuscript accordingly to enhance its quality.

I appreciate the analysis performed to evaluate the inter – variability. I am not sure that this test is appropriate to test it. Please revise this aspect with an expert statistician before the publication of the paper.
Response: We have now revised this aspect with the expert statistician of our department (Dr. Ujjwal Mukund Mahajan, PhD) to make sure our inter-variability test is valid.  He confirmed our analysis, we have now mentioned him in our acknowledgment. 

The authors reported that “In our study cohort, best-PEEP selection was handled by performing PEEP trials by arterial blood gas analysis at different PEEP levels”. This approach probably reflect the clinical practice but not reflect a good scientific sound. This aspect “We did not define best-PEEP by evaluating lung ultrasound when conducting this study” should be included in the main limitation of the study and please discuss the optimal approach taking into account the following papers in your discussion and add appropriate references ( doi: 10.1186/s13054-020-03311-9; doi: 10.1097/CCM.0000000000004640; doi: 10.1186/s40560-020-00499-4.)
Response: We have now added this information in the discussion of our manuscript as a main limitation of our study. Furthermore, we added a paragraph to discuss the optimal approach for defining best-PEEP, including your suggested references. 

Reviewer 3
The authors reponse well, however, I still have one minor suggestions.
Please delete the second paragraph in the discussion section because this is not the focus of this study. The discussion about lung ultrasound should be written first. 
Response: Thank you for this remark. We have now deleted the second paragraph in the discussion section and modified the order of the paragraphs.
