# Supplementary material for: Follow-up lung ultrasound to monitor lung failure in COVID-19 ICU patients
Source: PLoS One. 2022 Jul 14;17(7):e0271411. doi: 10.1371/journal.pone.0271411 (PMC9282475; doi:10.1371/journal.pone.0271411)
Supplement: Attachment — Submitted filename: Response to Reviewers.rtf [file pone.0271411.s005.rtf]

Dear Editor, Dear Editorial Board Members, Dear Reviewers, 
We thank the Reviewers for their input and have now adapted the manuscript accordingly. Please find a point-by-point response below.
Best regards, 
Michaela Barnikel and Stephanie-Susanne Stecher (on behalf of all co-authors)

Reviewer 1
The authors satisfied my previous comments. I suggest to improve the quality of the figure and i suggest to include a representative case. Please revise the figure in according with the guidelines of the journal. (delete the black)
I would like to congratulate with the authors for the efforts done to improve the quality of the paper.
Response:  Thank you for your previous comments and your efforts to improve our manuscript. We have now included a representative case to demonstrate the course of LUS and increased the resolution of our figures to improve their quality and to be in line with the guidelines of the journal. 
Reviewer 3
The authors response well, so I have no more comment. I recommend that the manuscript can be accepted now. 
Response: We thank the reviewer for his/her support.
